# dsRNA locks the door to viral movement

**DOI:** 10.1093/plcell/koad207

**Published:** 2023-07-24

**Authors:** Sara Lopez-Gomollon

**Affiliations:** Assistant Features Editor, The Plant Cell, American Society of Plant Biologists, USA; Department of Plant Sciences, University of Cambridge, Cambridge CB23EA, UK

Who has not caught the flu or chickenpox? We humans, and other organisms, are constantly challenged by pathogens and have evolved complex systems to fight back. Plants display 3 layers of defense: (1) an outer layer of physical and chemical barriers, (2) an inducible layer that triggers immunity by recognizing pathogen patterns at the cell surface (pattern-triggered immunity [PTI]) or pathogen effectors intracellularly (effector-triggered immunity) (Ngou et al. 2022), and (3), RNA silencing that counteracts exogenous viral RNA or DNA by producing small RNAs that target complementary viral sequences and regulates the other defense layers (Lopez-Gomollon and Baulcombe 2022).

An emerging picture is that RNA plays a pivotal role in plant–pathogen interactions. Apart from its primary role in RNA silencing, small RNAs and associated proteins can influence effector-triggered immunity and PTI, and these interactions can either reduce or reinforce virulence and defense (Lopez-Gomollon and Baulcombe 2022). It has also been shown that double-stranded (ds) RNA elicits antiviral defense through PTI independently of RNA silencing (Niehl et al. 2016). However, the underlying molecular mechanism and how viruses overcome this defense are unknown. In new work, **Caiping Huang, Ana Rocio Sede, Laura Elvira-González, and colleagues** (Huang et al. 2023) shed light on how dsRNA-induced PTI acts to inhibit virus infection and how viruses evade this plant host defense response.

The authors first investigated how dsRNA inhibits viral infection using *Nicotiana benthamiana* infected with a virus tagged with GFP. Treating the plants with dsRNA and visualizing the effect by in vivo fluorescence microscopy, the authors discovered that dsRNA did not affect viral replication or accumulation but inhibited viral cell-to-cell movement. Viruses exploit plasmodesmata—membrane channels that connect adjacent cells through cell walls enabling the transport of molecules between cells—to spread infection. The authors demonstrated that dsRNA inhibited viral movement by inducing plasmodesmata closure, achieved by increasing callose deposition in the cell wall region surrounding the channel.

In PTI, many pattern recognition receptors function with co-receptors to transduce downstream signals. A previous study showed that dsRNA elicits a PTI response mediated by a co-receptor in Arabidopsis (Niehl et al. 2016). Using in vivo imaging, plasmodesmata tagged lines, and PTI pathway mutants, the authors confirmed that in Arabidopsis, the same co-receptor is involved in callose deposition and that additional co-receptors also play a role. The authors further identified other PTI core components required for callose deposition at the plasmodesmata initiated by dsRNA. However, the PTI cascade triggered by dsRNA is independent of a reactive oxygen species burst, which is common in cascades triggered by other elicitors such as bacterial flagellin (Cheval et al. 2020). The authors concluded that the different PTI cascades triggered by dsRNA and other elicitors may have common components but also their unique signatures.

Viral movement proteins that evolved to counteract plant defenses are essential for mediating the movement of viruses. Using in vivo imaging and callose staining, the authors observed that movement proteins interfere with the dsRNA-induced callose deposition. They proposed a model whereby callose deposition at plasmodesmata is triggered by dsRNA to stop viral movement (see Figure). This mechanism may have evolved to isolate infected cells but still allows the virus to replicate. At the infection front, the virus prevents the closure of plasmodesmata to spread infection by the action of movement proteins. Behind the infection front, viral suppressors of RNA silencing are key to impairing RNA silencing and allowing for viral replication.

**Figure.**
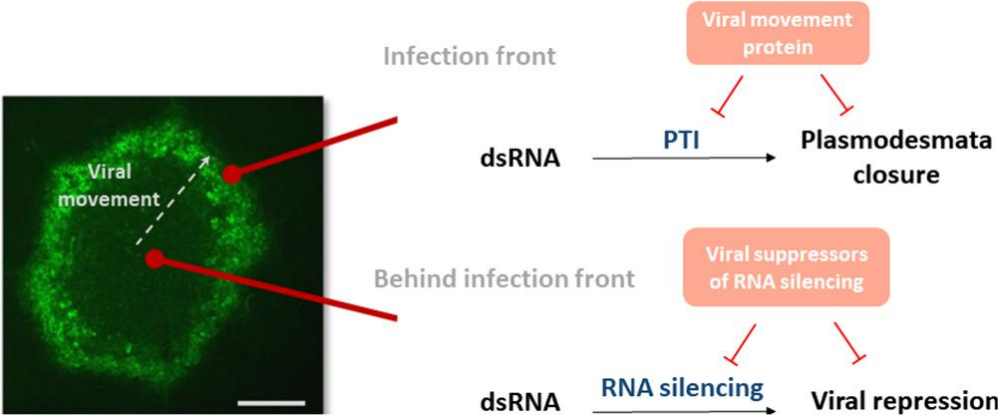
Model for plant–virus interaction during viral infection. dsRNA-triggered PTI and antiviral RNA silencing and both plant defence mechanism that may be suppressed by viral effector proteins to support virus propagation and replication. Viral movement proteins act in cells at the virus infection front to facilitate virus movement by blocking a dsRNA-induced callose defence response at plasmodesmata. Viral suppressors of RNA silencing block dsRNA-induced antiviral RNA silencing in the center of infection sites to support virus replication. Left image: A local infection site of tobacco mosaic virus encoding a movement protein fused to GFP 7 days after infection in *Nicotiana benthamiana*. White arrow shows viral movement direction. Scale bar, 1 mm. Adapted from Huang et al. (2023), Figure 10C.

The work of Huang, Sede, Elvira-González, and colleagues (Huang et al. 2023) is an outstanding contribution to our understanding of viral infection and plant response. For example, the results suggest that viral movement proteins do not open plasmodesmata but prevent their closure triggered by an immune response, explaining why the presence of movement proteins from one virus increases susceptibility to other viruses (Cooper et al. 1995). This research also raises interesting questions, such as the identity of the dsRNA receptor and other missing components of the PTI pathway, the detailed mechanism of how movement proteins interfere with plasmodesmata closure, and the balance between RNA silencing and dsRNA-triggered PTI response during infection. Exciting times are ahead in the field of plant defense!

## References

[koad207-B1] Cheval C , Samwald S, Johnston MG, de Keijzer J, Breakspear A, Liu X, Bellandi A, Kadota Y, Zipfel C, Faulkner C. Chitin perception in plasmodesmata characterizes submembrane immune-signaling specificity in plants. Proc Natl Acad Sci U S A. 2020:117(17):9621–9629. 10.1073/pnas.1907799117 32284410 PMC7196898

[koad207-B2] Cooper B , Lapidot M, Heick JA, Allan Dodds J, Beachy RN. A defective movement protein of TMV in transgenic plants confers resistance to multiple viruses whereas the functional analog increases susceptibility. Virology. 1995:206(1):307–313. 10.1016/S0042-6822(95)80046-8 7831786

[koad207-B3] Huang C , Sede AR, Elvira-González L, Yan Y, Rodriguez M, Mutterer J, Boutant E, Shan L, Heinlein M. dsRNA-induced immunity targets plasmodesmata and is suppressed by viral movement proteins. Plant Cell. 2023:35(10):3845–3869. 10.1093/plcell/koad176 PMC10533371 37378592

[koad207-B4] Lopez-Gomollon S , Baulcombe DC. Roles of RNA silencing in viral and non-viral plant immunity and in the crosstalk between disease resistance systems. Nat Rev Mol Cell Biol. 2022:23(10):645–662. 10.1038/s41580-022-00496-5 35710830

[koad207-B5] Ngou BPM , Jones JDG, Ding P. Plant immune networks. Trends Plant Sci. 2022:27(3):255–273. 10.1016/j.tplants.2021.08.012 34548213

[koad207-B6] Niehl A , Wyrsch I, Boller T, Heinlein M. Double-stranded RNAs induce a pattern-triggered immune signaling pathway in plants. New Phytol. 2016:211(3):1008–1019. 10.1111/nph.13944 27030513

